# Yield and Quality in Main and Ratoon Crops of Grain Sorghum Under Different Nitrogen Rates and Planting Densities

**DOI:** 10.3389/fpls.2021.778663

**Published:** 2022-01-13

**Authors:** Yu Zhou, Juan Huang, Zebi Li, Yu Wu, Jijun Zhang, Yaqin Zhang

**Affiliations:** Institute of Characteristic Crops Research, Chongqing Academy of Agricultural Sciences, Chongqing, China

**Keywords:** grain sorghum, ratooning, yield, quality, nitrogen fertilizer, planting density

## Abstract

Ratooning is the cultivation practice of two harvests in one cropping season by producing a second crop from the original stubble, which could provide higher resource use efficiency and economic benefit compared with direct sown crops. Nitrogen (N) fertilizer and planting density (D) play a vital role in sorghum (*Sorghum bicolor* L.) production; however, limited information is available on the effects on yield and quality of the sorghum-ratoon system. To address this question, field experiments were conducted with three N treatments (120 kg N ha^–1^, N1; 180 kg N ha^–1^, N2; and 255 kg N ha^–1^, N3) and three D treatments (82,500 plant ha^–1^, D1; 105,000 plant ha^–1^, D2; and 127,500 plant ha^–1^, D3). The yield of the main crop was significantly higher than that of the ratoon crop. Increasing N could increase the yield and yield attributes of both main and ratoon crops, and the effect on the ratoon crop was greater than the main crop. With increasing D, the grain yield of both main and ratoon crops increased, though 1,000-grain weight and grain weight per ear decreased. The sorghum grain of the ratoon crop contained higher starch, protein, and tannin contents but lower fat content, indicating a better quality for liquor production. The quality traits were significantly affected by N and D, but the differences between treatments were smaller than that between the main and ratoon crop. Our results indicated that increasing the yield of ratoon crops could obtain a high yield and quality of the sorghum-ratoon system. It was recommended that 120 kg N ha^–1^ with 127,500 plant ha^–1^ for the main crop and a small amount of N be top-dressed in three new buds left on stubble in each hill for the ratoon crop.

## Introduction

Sorghum, the fifth most important cereal crop in the world with high-stress tolerance and wide adaptability, is grown for both human food and animal feed. In China, sorghum is planted in an area of about 751,793 hectares, produces about 3.60 million tons of grains^1^; this places China at 12th and 6th position in the world, respectively. Waxy sorghum is used as the main raw material for the production of liquor (Ni et al., 2015); about 80% of the domestic production in China is used for this purpose (Wang C. et al., 2016). In recent years, the demand for sorghum has increased substantially due to the rapid increase in the liquor industry in China. Due to the shortfall of domestic supply, a lot of the Chinese industries are producing liquor by using imported sorghum. Most of the imported sorghum is of forage quality; the use of this type reduces the quality of the liquor (Zou et al., 2020). The climate in southwest China is suitable for the production of waxy sorghum (Lu et al., 2009), and this can provide raw material to the liquor industries in China (Wang C. et al., 2017).

Ratooning of sorghum is often practiced in different parts of the world where a second crop is harvested in the same cropping season. In a ratoon crop, the basal buds of the stem grow shortly after cutting the main crop (Wilson, 2011). Normally, the duration of a ratoon crop is much shorter than the direct seeded crop (Al-Taweel et al., 2020), providing higher resource use efficiency per unit time and per unit land area (Santos et al., 2003). Therefore, ratooning of the sorghum crop is widely practiced in this region where photothermal resources exceed the demand of single-season sorghum but are insufficient for double-season sorghum (Yin et al., 2015). Ratooning has several advantages, such as there is no need for land preparation and new seed, and it also eliminates the risk of any seeding delay and other risks associated with crop establishment (Escalada and Plucknett, 1975; Gerik et al., 1988).

The yield of a ratoon crop can be increased through the identification and the use of appropriate management practices (Santos et al., 2003; Petroudi et al., 2011; Rogé et al., 2016). Of the different management practices, the application of nitrogen (N) fertilizer and planting density (D) play the most important roles for getting higher grain yield and better quality in the ratoon crops. N fertilizer enhances the formation of tillers and thus gives greater yield (Escalada and Plucknett, 1977). Often, a higher rate of N fertilization gives greater forage, dry matter, and grain yield (Mahfouz et al., 2015); however, grain yield does not increase linearly after a certain rate (Touchton and Martin, 1981). Some studies on N management have been carried out on both main and ratoon sorghum (Rao et al., 2011). In most cases, surplus N was found in soil after meeting the demand of the main crop (He et al., 2016). According to the study by Ceotto et al. (2014), it is not worthwhile to fertilize sorghum under good soil fertility conditions. It was widely confirmed that N fertilization affected the seed quality traits of both grain and sweet sorghum (Rashid et al., 2008; Tang et al., 2018). However, less information is available about the application of N fertilizers on the quality of the waxy sorghum, much less on the main and ratoon sorghum.

Plant density can affect the plant and canopy architecture of the crop and thus can influence the number of effective ear per unit area, leaf area index (LAI), and radiation interception (Westgate et al., 1997; Tabo et al., 2002). Plant density can also affect the growth of the crop, grain yield and its quality (Defoor et al., 2001; Carmi et al., 2006). Compared with the main crop, ratoon sorghum becomes taller but produces lower biomass yield (Vinutha et al., 2017); therefore, under the same D, ratoon crop may build a different population structure. To our knowledge, no literature is available on the effect of D on the main and ratoon crop of waxy sorghum. The objective of this study was to understand the effects of N fertilizer and D on the main and ratoon crop for yield and quality traits of waxy sorghum.

## Materials and Methods

### Site Description

A 2-year field experiment was conducted in 2019 and 2020 at the Yuxi Crop Experimental Station of the Chongqing Academy of Agricultural Sciences, Yongchuan District (105.71°E longitude, 29.75°N latitude, 298 m altitude a.s.l.), Chongqing, China. Soil samples from the upper 20 cm were collected for soil analysis before seeding in 2019. The soil was in a clay loam texture with a pH of 4.5, organic matter content of 23.9 g kg^–1^, total N of 0.76 g kg^–1^, available N of 98.3 mg kg^–1^, available P of 13.4 mg kg^–1^, and available K of 102.7 mg kg^–1^. The climate of this county is subtropical humid monsoon with an annual average temperature of 17.7°C, a maximum temperature of 42.1°C, and a minimum temperature of –2.9°C. The frost-free period is about 317 days, and the annual mean precipitation is about 1015.0 mm.

### Experimental Design and Treatments

A waxy semidwarf sorghum hybrid cultivar “Jinyunuo 3” was used in this study. This cultivar is well adapted in southwest China, possessing high yield potential, good seed quality properties, and strong ratooning ability. The experiments were laid out in a split-plot design with N rates as the main plot and D as a subplot, and the number of replications was three.

The fertilizer urea containing 46.4% N was used as the source of N. The 3 N doses were as follows: 120 (N1), 180 (N2), and 255 (N3) kg ha^–1^ and were applied at two stages in the main crop at complete field emergence and at the jointing stage, in a ratio of 3:7; no N fertilizer was applied to the ratoon crop. The D treatments were 82,500 (D1), 105,000 (D2), and 127,500 (D3) plant ha^–1^. The plot size was 6-m long × 5-m wide with 12 rows; row spacing was 50 cm. The hill seeding was performed, where the distance between the hills was 48.5 cm for D1, 38.1 cm for D2, and 31.4 cm for D3. The crop was seeded on March 21, 2019, and on April 4, 2020; seedlings were thinned at the five-leaf stage leaving two plants at each hill. The main crop was harvested by reaping ears manually on July 26, 2019, and August 6, 2020, followed by cutting the stem at 5–10 cm above the ground. After new tillers on stubble regenerated, one of them was left in each plant and allowed to grow to maturity. The ratoon sorghum was harvested on November 18, 2019, and November 19, 2020.

### Growth Parameters and Dry Weight

Three plants from each plot were selected randomly and were used to measure LAI and chlorophyll content at flowering and mature stages in both seasons. The LAI was calculated as the total green leaf area divided by the harvest area of the plot. The green leaf area (cm^2^) of the individual leaves was calculated using the formula as follows: Leaf length (cm) × maximum width of a leaf (cm) × 0.75. The chlorophyll content (soil-plant analysis and development, SPAD) was measured on the second leaf from the top of the plant using the SPAD-502 Plus device (Konica Minolta, Japan).

In both seasons, three representative plants were sampled from each plot at the mature stage to collect the data of both plant height (cm, PH) and stem diameter (cm, SD). PH was measured from the soil surface to the top of the panicle by a telemeter rod, and SD was measured at the second internode from the stem base by a vernier caliper. The plants were cut at the ground level, chopped, and dried in an oven at 80°C for at least 4 days to obtain aboveground total dry weight (TDW).

### Yield and Yield Components

A random sample of 10 plants from each plot was harvested at maturity and was threshed manually to measure grain weight per ear (g, GWPE) and 1,000-grain weight (g, TGW); the remaining plants were harvested to estimate plot yield and data converted to kg per ha. Grain moisture content was determined immediately after threshing using a Riceter Grain Moisture Meter (Kett Electric Laboratory, Tokyo, Japan). The TGW, GWPE, and yield were reported at a moisture content of 130 g H_2_O kg^–1^ fresh weight.

### Grain Quality

Starch, protein, fat, and tannin contents of the seed samples were determined by using a DA 7250 near-IR grain quality analysis meter (Perten, Sweden). For this, three subsamples (small sample plate 44 cm^2^) from each plot were used (Qu et al., 2019), and the mean values were used for statistical analysis.

### Calculations and Data Analysis

The ANOVA was performed for each year on all traits using Statistical Product and Service Solutions (SPSS, version 19.0; IBM SPSS Inc. Chicago, IL, United States). The effects of the growing season (S; main and ratoon seasons), N, D, and their interactions on the traits were analyzed following the generalized linear model procedure. Tests for significant differences between the treatments were carried out using Duncan’s multiple range tests at a 5% level of significance.

## Results

### Weather Conditions

**FIGURE 1:**
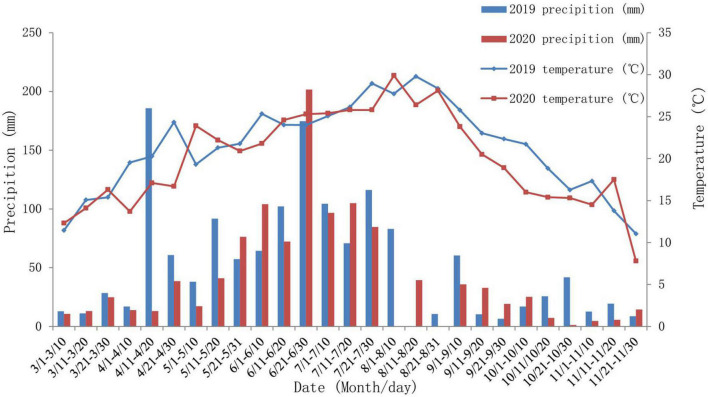
Precipitation and mean temperature during the growth period (2019–2020) of the sorghum-ratoon system at the experimental site, Yongchuan County, Chongqing City, southwest China.

The precipitation and temperature at the experimental site during the crop growing periods were collected from a nearby meteorological station and are shown in Figure 1. Total precipitation during the growing season was 1,430.6 mm in 2019 and 1,097.6 mm in 2020. The accumulated precipitations during the main and ratoon seasons were higher in 2019 than in 2020, which was due to two heavy rainfalls in mid-April and late June in 2019.

The mean temperature was 21.4°C in 2019 and 20.0°C in 2020. The temperature increased across the main season and decreased over the ratoon season. The average temperatures during the main and ratoon seasons were both higher in 2019 than those in 2020.

### Growth Parameters and Dry Weight

The SPAD was significantly affected by S, N, and D but not by their interactions except the S × N interaction (Table 1). The LAI was significantly affected by S, N, D, and their interactions in 2019, but in 2020, only LAI in the mature stage was affected by S × N, S × D, and N × D interactions (Table 1).

**TABLE 1:** Leaf area index (LAI) and soil-plant analysis and development (SPAD) at flowering and mature stage of the main and ratoon crops for different treatments in 2019 and 2020.

Treatment	2019				2020			
	SPAD		LAI		SPAD		LAI	
	Flowering	Mature	Flowering	Mature	Flowering	Mature	Flowering	Mature
Main crop								
N1	53.07 ± 2.47^a^	49.39 ± 1.57^b^	5.28 ± 0.57^c^	4.93 ± 0.82^c^	53.20 ± 1.74^b^	50.53 ± 1.99^c^	5.16 ± 0.89^c^	4.89 ± 0.82^c^
N2	54.23 ± 2.47^a^	50.70 ± 1.25^b^	5.67 ± 0.86^b^	5.11 ± 0.88^b^	54.89 ± 2.90^a^	52.39 ± 1.94^b^	5.81 ± 1.00^b^	5.23 ± 0.93^b^
N3	55.44 ± 2.25^a^	52.74 ± 2.19^a^	6.04 ± 0.90^a^	5.23 ± 0.85^a^	55.66 ± 2.71^a^	53.72 ± 1.84^a^	6.15 ± 0.93^a^	5.35 ± 0.91^a^
D1	56.50 ± 1.26^a^	52.24 ± 2.00^a^	4.94 ± 0.32^c^	4.05 ± 0.12^c^	57.27 ± 2.16^a^	54.20 ± 1.70^a^	4.65 ± 0.54^c^	4.06 ± 0.19^c^
D2	54.45 ± 1.56^b^	50.30 ± 2.08^a^	5.40 ± 0.22^b^	5.23 ± 0.18^b^	54.24 ± 1.41^b^	51.94 ± 1.43^b^	5.71 ± 0.29^b^	5.35 ± 0.21^b^
D3	51.80 ± 1.98^b^	50.30 ± 2.03^a^	6.65 ± 0.55^a^	5.99 ± 0.15^a^	52.24 ± 1.08^c^	50.49 ± 2.01^c^	6.77 ± 0.56^a^	6.06 ± 0.32^a^
Mean	54.25 ± 2.51	50.95 ± 2.17	5.66 ± 0.82	5.09 ± 0.83	54.58 ± 2.62	52.21 ± 2.28	5.71 ± 1.00	5.16 ± 0.88
Ratoon crop								
N1	48.30 ± 2.39^a^	39.81 ± 2.65^c^	4.59 ± 0.42^c^	1.27 ± 0.25^c^	47.59 ± 2.97^c^	39.77 ± 1.95^c^	4.42 ± 0.47^c^	1.52 ± 0.09^c^
N2	50.50 ± 2.12^a^	44.26 ± 2.19^b^	5.24 ± 0.55^b^	2.17 ± 0.52^b^	50.10 ± 1.73^b^	43.13 ± 2.71^b^	4.96 ± 0.69^b^	2.01 ± 0.34^b^
N3	51.73 ± 2.30^a^	48.60 ± 2.99^a^	5.57 ± 0.30^a^	2.59 ± 0.32^a^	52.60 ± 2.45^a^	49.74 ± 2.53^a^	5.39 ± 0.39^a^	2.35 ± 0.23^a^
D1	52.32 ± 1.86^a^	44.87 ± 5.10^a^	4.73 ± 0.46^c^	1.66 ± 0.51^b^	52.41 ± 2.16^a^	45.62 ± 5.03^a^	4.37 ± 0.42^c^	1.73 ± 0.35^c^
D2	49.82 ± 2.36^b^	45.23 ± 3.20^a^	5.07 ± 0.57^b^	2.25 ± 0.58^a^	50.05 ± 2.29^b^	45.26 ± 4.81^a^	4.92 ± 0.56^b^	1.98 ± 0.36^b^
D3	48.39 ± 2.08^c^	42.57 ± 4.83^b^	5.59 ± 0.42^a^	2.11 ± 0.81^a^	47.84 ± 3.21^c^	41.77 ± 4.12^b^	5.48 ± 0.44^a^	2.16 ± 0.46^a^
Mean	50.18 ± 2.62	44.22 ± 4.45	5.13 ± 0.59	2.01 ± 0.67	50.10 ± 3.14	44.22 ± 4.82	4.93 ± 0.65	1.96 ± 0.42
*F*-value								
S	107.85**	146.76**	166.49**	6108.66**	140.03**	381.77**	347.36**	10218.31**
N	9.92*	54.06**	196.32**	87.05**	51.17**	362.20**	108.09**	97.09**
D	40.48**	4.98*	336.47**	332.98**	53.75**	29.38**	496.05**	501.18**
S × N	0.80	8.05**	3.73*	59.31**	3.95*	25.25**	0.60	11.67**
S × D	0.82	2.26	41.25**	119.47**	0.27	2.59	48.36515506	213.21**
N × D	0.39	1.18	5.65**	7.17**	0.52	0.36	5.594269507	7.22**
S × N × D	0.32	0.38	7.60**	4.98**	1.79*	0.78	7.559578395	1.00

For each treatment (N or D), the lowercase letter in the same column indicates significant differences at 0.05 level. *Significant at the 0.05 probability level.

**Significant at the 0.01 probability level.

The SPAD and LAI of the main crop were significantly higher than those of the ratoon crop. Both SPAD and LAI decreased at the mature stage as compared to the flowering stage in both main and ratoon crops, and this decrease was more pronounced in the ratoon crop (Table 1). On average, the SPAD decreased by 5.21% at the mature stage compared with that at the flowering stage for the main crop but 11.81% for the ratoon crop. LAI at the mature stage was lower than that at the flowering stage by 9.87% for the main crop but 60.56% for the ratoon crop.

For both main and ratoon crops, the SPAD and LAI at flowering and mature stages increased with increasing N rate. With the increment of D, at flowering and mature stages, SPAD declined, while LAI increased, in both growing seasons.

The ANOVA results (Table 2) showed that the PH was significantly affected by S and N in both years and the interactions of S × N and S × D in 2020. The SD was significantly affected by S, N, and D but not by their interactions. The TDW was significantly affected by S, N, and D in both years and the interactions of S × D and N × D in 2019. The PH, SD, and TDW of the main crop were 17.18, 12.47, and 46.64% higher than the ratoon crop over 2 years. With the increment of N rates, the PH, SD, and TDW of the main and ratoon crops increased in 2019 and 2020. In case of the treatment D, no difference for PH was found in both main and ratoon crops in 2019; however, in 2020, the PH increased in the main crop but decreased in the ratoon crop with increasing D treatment. In both crops, the SD and TDW decreased with increasing D in 2019 and 2020.

**TABLE 2:** The plant height (PH), stem diameter (SD), and total dry weight (TDW) of the main and ratoon crops for different treatments in 2019 and 2020.

Treatment	2019			2020		
	PH (cm)	SD (mm)	TDW (g plant^–1^)	PH (cm)	SD (mm)	TDW (g plant^–1^)
Main crop						
N1	204.89 ± 4.01^b^	18.53 ± 1.67^b^	169.41 ± 8.53^c^	204.67 ± 6.6^b^	20.03 ± 1.41^b^	178.88 ± 10.36^c^
N2	209.78 ± 4.15^a^	19.84 ± 1.54^ab^	178.41 ± 6.08^b^	214.56 ± 8.02^a^	20.77 ± 1.31^b^	193.70 ± 10.55^b^
N3	209.22 ± 4.18^a^	20.96 ± 1.86^a^	185.77 ± 7.36^a^	218.11 ± 5.35^a^	22.55 ± 1.15^a^	204.92 ± 12.73^a^
D1	205.22 ± 5.04^a^	21.03 ± 1.62^a^	183.53 ± 7.74^a^	208.33 ± 8.00^b^	21.93 ± 1.43^a^	202.55 ± 14.15^a^
D2	208.67 ± 3.77^a^	20.09 ± 1.42^a^	177.91 ± 7.50^b^	212.11 ± 7.69^ab^	21.40 ± 1.47^a^	194.26 ± 11.98^b^
D3	210.00 ± 3.67^a^	18.21 ± 1.62^b^	172.14 ± 11.21^c^	216.89 ± 9.01^a^	20.02 ± 1.56^b^	180.70 ± 12.12^c^
Mean	207.96 ± 4.54	19.78 ± 1.92	177.86 ± 9.84	212.44 ± 8.69	21.12 ± 1.65	192.50 ± 15.34
Ratoon crop						
N1	173.33 ± 7.18^a^	16.87 ± 0.96^b^	89.08 ± 10.90^c^	171.44 ± 4.03^b^	17.23 ± 0.68^b^	85.33 ± 9.99^c^
N2	175.11 ± 4.70^a^	17.52 ± 1.41^b^	99.28 ± 13.80^b^	172.89 ± 3.37^ab^	17.84 ± 1.31^b^	97.37 ± 9.98^b^
N3	176.00 ± 7.31^a^	18.57 ± 1.55^a^	112.28 ± 13.15^a^	175.78 ± 3.19^a^	19.34 ± 0.86^a^	109.57 ± 8.02^a^
D1	174.67 ± 9.22^a^	19.02 ± 1.30^a^	114.21 ± 13.01^a^	175.11 ± 2.98^a^	18.78 ± 1.24^a^	106.01 ± 9.78^a^
D2	175.11 ± 4.96^a^	17.18 ± 1.09^b^	99.34 ± 7.75^b^	174.56 ± 3.97^a^	18.27 ± 1.10^ab^	98.23 ± 12.91^b^
D3	174.67 ± 4.64^a^	16.76 ± 0.92^b^	87.08 ± 11.91^c^	170.44 ± 3.09^b^	17.37 ± 1.30^b^	88.04 ± 12.21^c^
Mean	174.81 ± 6.36	17.66 ± 1.46	100.21 ± 15.55	173.37 ± 3.87	18.14 ± 1.31	97.43 ± 13.52
*F*-value						
S	355.44**	45.84**	4229.51**	842.43**	119.08**	3014.96**
N	30.07**	27.29**	84.55**	17.09**	89.37**	86.36**
D	0.70	22.02**	86.84**	0.79	12.84**	44.62**
S × N	0.26	0.550	3.120	4.74*	0.190	0.220
S × D	0.63	1.87	14.63**	8.36**	0.36	0.49
N × D	0.02	0.80	4.43**	0.63	0.31	0.07
S × N × D	0.19	0.17	2.01	0.63	0.29	0.86

*For each treatment (N or D), the lowercase letter in the same column indicates significant differences at 0.05 level.*

*Significant at the 0.05 probability level. **Significant at the 0.01 probability level.

### Yield and Yield Components

The ANOVA results (Table 3) showed that S, N, and D had highly significant effects on the TGW, GWPE, and yield, and the interaction of S × N had a significant effect on yield in both years and the GWPE in 2020, and the interaction of S × D had a significant effect on yield in both years. The TGW, GWPE, and yield of the main crop were significantly higher than those of the ratoon crop by 26.39, 50.62, and 41.86% in 2019 and 34.51, 56.27, and 50.38% in 2020. It indicated that the yield of the main crop was significantly higher than that of the ratoon crop mainly due to more GWPE. The yield increased with increasing N, and the increment of the ratoon crop was greater than that of the main crop (Table 3). The yield of the main crop under N3 was higher than those under N1 and N2 by 7.92 and 3.60% in 2019 and by 8.83 and 2.41% in 2020. However, the yield of the ratoon crop under N3 was higher than those under N1 and N2 by 35.16 and 20.91% in 2019 and by 45.68 and 20.88% in 2020. The greatest total yield of main and ratoon sorghum was obtained under N3, which was 17.27 and 9.82% higher than those under N1 and N2 in 2019 and 19.73 and 6.75% in 2020. The TGW and GWPE of the main and ratoon crops increased with increasing N, and the increments of the ratoon crop were greater than those of the main crop. Averaged across years, the TGW under N3 was 5.63 and 2.15% higher than those under N1 and N2 in the main season, while those were 7.12 and 3.98% in the ratoon season. On average, the N3 treatment produced 14.02 and 7.47% higher GWPE than N1 and N2 in the main crop and 37.35 and 23.24% in the ratoon crop. It could be inferred that higher yield under a high N rate was mainly composed of increasing GWPE. Comparing the positive effects of N on productivity parameters of main and ratoon crops, N had a larger effect on the grain yield of ratoon crops.

**TABLE 3:** Grain yield and yield components of the main and ratoon crops for different treatments in 2019 and 2020.

Treatment	2019			2020		
	TGW (g)	GWPE (g)	Yield (kg ha^–1^)	TGW (g)	GWPE (g)	Yield (kg ha^–1^)
Main crop						
N1	25.22 ± 1.45^b^	56.33 ± 4.90^c^	5038.95 ± 588.42^c^	26.67 ± 1.23^b^	59.28 ± 3.26^c^	5369.04 ± 437.58^c^
N2	25.75 ± 1.08^ab^	59.92 ± 4.58^b^	5249.28 ± 488.69^b^	27.91 ± 1.13^a^	62.75 ± 3.34^b^	5705.27 ± 383.62^b^
N3	26.51 ± 0.96^a^	65.03 ± 5.06^a^	5438.05 ± 514.23^a^	28.30 ± 1.18^a^	66.79 ± 3.38^a^	5843.01 ± 348.32^a^
D1	27.04 ± 0.91^a^	65.69 ± 4.07^a^	4653.28 ± 237.87^c^	28.86 ± 0.96^a^	66.65 ± 3.59^a^	5183.84 ± 263.11^c^
D2	25.63 ± 0.96^b^	59.99 ± 4.56^b^	5232.57 ± 220.89^b^	27.41 ± 0.75^b^	62.80 ± 3.01^b^	5679.49 ± 245.90^b^
D3	24.80 ± 0.73^c^	55.60 ± 4.32^c^	5840.43 ± 202.22^a^	26.61 ± 1.18^b^	59.36 ± 3.62^c^	6053.99 ± 188.06^a^
Mean	25.82 ± 1.26	60.43 ± 5.91	5242.09 ± 537.56	27.63 ± 1.34	62.94 ± 4.47	5639.11 ± 427.41
Ratoon crop						
N1	18.46 ± 1.43^b^	25.96 ± 3.14^c^	2634.72 ± 273.84^c^	17.43 ± 1.25^a^	23.38 ± 2.58^c^	2292.39 ± 264.61^c^
N2	19.11 ± 1.01^a^	28.53 ± 4.50^b^	2945.12 ± 287.71^b^	17.86 ± 1.50^a^	26.46 ± 3.51^b^	2762.82 ± 318.82^b^
N3	19.45 ± 1.31^a^	35.03 ± 4.04^a^	3561.01 ± 406.19^a^	18.99 ± 1.14^a^	32.73 ± 3.04^a^	3339.58 ± 319.53^a^
D1	20.52 ± 0.67^a^	33.85 ± 4.64^a^	2656.10 ± 323.19^c^	19.43 ± 0.84^a^	30.49 ± 4.43^a^	2442.83 ± 457.91^c^
D2	18.54 ± 0.56^b^	30.09 ± 4.40^b^	3164.39 ± 532.76^b^	17.81 ± 1.05^b^	27.97 ± 4.57^b^	2874.51 ± 454.90^b^
D3	17.95 ± 0.72^c^	25.58 ± 4.02^c^	3320.35 ± 403.90^a^	17.04 ± 1.18^c^	24.11 ± 3.96^c^	3077.45 ± 489.66^a^
Mean	19.01 ± 1.28	29.84 ± 5.43	3046.95 ± 503.19	18.09 ± 1.42	27.52 ± 4.94	2798.26 ± 524.11
*F*-value						
S	1576.77**	3057.18**	3698.19**	1988.50**	14863.10**	8626.65**
N	52.04**	69.46**	96.27**	34.48**	102.33**	132.21**
D	69.04**	91.88**	221.45**	40.84**	184.94**	205.34**
S × N	0.53	0.56	20.06**	1.47	5.63**	32.03**
S × D	0.94	1.29	20.58**	0.06	1.82	5.29*
N × D	1.72	0.45	1.41	0.70	1.51	0.12
S × N × D	0.68	0.22	1.57	0.62	1.30	0.92

*For each treatment (N or D), the lowercase letter in the same column indicates significant differences at 0.05 level.*

*Significant at the 0.05 probability level. **Significant at the 0.01 probability level.

With increasing D, the grain yield of both main and ratoon crops increased, while TGW and GWPE decreased (Table 3). D3 treatment produced more grain yield of the main crop than D1 and D2 by 25.51 and 11.62% in 2019 and 16.79 and 6.59% in 2020. However, the ratoon sorghum under D3 had 25.01 and 4.93% more yield than those under D1 and D2 in 2019 and 25.98 and 7.06% in 2020. D3 treatment produced more total yield than D1 and D2 by 25.01 and 4.93% in 2019 and 19.73 and 6.75% in 2020. Averaged across years, 5.39 and 8.75% more TGW were caused by D1 than those by D2 and D3 in the main crop, while 9.92 and 14.18% were in the ratoon crop. On average, the D1 treatment induced 7.78 and 15.13% higher GWPE than D2 and D3 in the main crop and 10.83 and 29.49% in the ratoon crop. The reductions of TGW and GWPE from D1 to D3 in the ratoon crop were greater than those in the main crop. Although the TGW and GWPE both decreased with increasing D, the yield still continued to increase, inferring that the main reason for higher yield under high density was the increase of effective panicle number per unit area.

Comparing the effects of N and D on the sorghum yield, it could be concluded that N was more efficient than D on the productivity parameters of the ratoon crop, while D was more efficient in the main season.

### Grain Quality

The ANOVA results (Table 4) showed that the starch content was significantly affected by S in 2019 and S, N, and D in 2020 and by the interactions of S × N and S × D in both years. The protein content was significantly affected by S, N, D, and S × D interaction in both years. The fat content was significantly affected by S and N in both years and by S × D interaction in 2019 and D in 2020. The tannin content was significantly affected by S, N, D, and S × D interaction in both years and by the interactions of S × N and N × D in 2020. The grain starch, protein, and tannin contents of the ratoon crop were 3.02, 6.80, and 141.63% higher than those of the main crop in 2019 and 3.42, 8.87, and 151.48% in 2020. The ratoon crop contained a lower grain fat content than the main crop by 9.24% in 2019 and 7.64% in 2020.

**TABLE 4:** Grain quality traits of the main and ratoon crops for different treatments in 2019 and 2020.

Treatment	2019				2020			
	Starch (%)	Protein (%)	Fat (%)	Tannin (%)	Starch (%)	Protein (%)	Fat (%)	Tannin (%)
Main crop								
N1	73.56 ± 0.98^a^	9.12 ± 0.28^c^	3.87 ± 0.12^a^	0.36 ± 0.02^b^	73.32 ± 1.13^a^	8.67 ± 0.19^c^	3.90 ± 0.26^a^	0.37 ± 0.02^b^
N2	72.88 ± 0.65^a^	9.28 ± 0.32^b^	3.69 ± 0.11^b^	0.36 ± 0.03^b^	72.99 ± 0.54^a^	9.02 ± 0.25^b^	3.77 ± 0.18^a^	0.38 ± 0.06^ab^
N3	71.60 ± 0.89^b^	9.60 ± 0.48^a^	3.54 ± 0.11^c^	0.40 ± 0.04^a^	71.09 ± 0.68^b^	9.26 ± 0.24^a^	3.55 ± 0.19^b^	0.40 ± 0.03^a^
D1	72.13 ± 1.21^a^	8.87 ± 0.15^b^	3.78 ± 0.19^a^	0.33 ± 0.01^c^	73.02 ± 1.68^a^	8.78 ± 0.29^c^	3.88 ± 0.17^a^	0.36 ± 0.02^b^
D2	72.79 ± 1.21^a^	9.58 ± 0.30^a^	3.65 ± 0.20^b^	0.38 ± 0.02^b^	72.41 ± 1.02^ab^	9.20 ± 0.35^a^	3.72 ± 0.32^ab^	0.37 ± 0.01^b^
D3	73.12 ± 0.95^a^	9.55 ± 0.27^a^	3.67 ± 0.14^b^	0.40 ± 0.03^a^	71.97 ± 0.90^b^	8.98 ± 0.21^b^	3.63 ± 0.18^b^	0.42 ± 0.04^a^
Mean	72.68 ± 1.16	9.34 ± 0.41	3.70 ± 0.18	0.37 ± 0.04	72.47 ± 1.28	8.99 ± 0.33	3.74 ± 0.25	0.38 ± 0.04
Ratoon crop								
N1	74.61 ± 0.99^b^	9.80 ± 0.22^c^	3.49 ± 0.16^a^	0.86 ± 0.04^a^	73.89 ± 0.60^c^	9.55 ± 0.33^c^	3.53 ± 0.15^a^	0.91 ± 0.02^b^
N2	74.01 ± 0.42^b^	9.95 ± 0.13^b^	3.37 ± 0.15^a^	0.90 ± 0.03^a^	75.04 ± 0.86^b^	9.80 ± 0.42^b^	3.47 ± 0.14^a^	0.94 ± 0.02^b^
N3	76.00 ± 0.85^a^	10.16 ± 0.16^a^	3.22 ± 0.18^b^	0.92 ± 0.02^a^	75.90 ± 0.79^a^	10.01 ± 0.33^a^	3.37 ± 0.10^a^	1.03 ± 0.07^a^
D1	75.17 ± 1.28^a^	10.11 ± 0.17^a^	3.20 ± 0.20^b^	0.91 ± 0.04^a^	74.80 ± 0.88^b^	9.90 ± 0.19^b^	3.46 ± 0.05^a^	0.98 ± 0.10^a^
D2	75.11 ± 1.04^a^	10.01 ± 0.13^b^	3.45 ± 0.12^a^	0.89 ± 0.04^a^	75.50 ± 1.26^a^	10.11 ± 0.25^a^	3.52 ± 0.19^a^	0.95 ± 0.04^a^
D3	74.35 ± 1.03^a^	9.78 ± 0.23^c^	3.42 ± 0.16^a^	0.90 ± 0.03^a^	74.53 ± 1.05^b^	9.34 ± 0.24^c^	3.39 ± 0.15^a^	0.95 ± 0.04^a^
Mean	74.87 ± 1.14	9.97 ± 0.23	3.36 ± 0.19	0.90 ± 0.04	74.94 ± 1.11	9.78 ± 0.40	3.46 ± 0.14	0.96 ± 0.07
*F*-value								
S	119.99**	525.29**	120.64**	6349.11**	167.49**	610.00**	47.41**	5734.05**
N	2.65	79.89**	31.15**	17.60**	6.99*	73.47**	23.20**	14.24*
D	0.76	39.89**	1.61	6.92**	5.66**	81.13**	5.07*	4.28*
S × N	30.29**	2.12	0.51	1.960	42.34**	1.460	1.790	12.33**
S × D	6.83**	124.04**	14.07**	13.29**	3.98*	49.94**	2.48	12.47**
N × D	1.64	2.53	0.65	0.40	1.92	1.85	2.66	3.61*
S × N × D	0.25	4.46**	0.96	1.47	0.49	1.40	0.14	5.30**

*For each treatment (N or D), the lowercase letter in the same column indicates significant differences at 0.05 level.*

*Significant at the 0.05 probability level. **Significant at the 0.01 probability level.

With increasing N rate, the starch content decreased in the main crop, while increased in the ratoon crop with increasing N rate. The protein and tannin contents of both main and ratoon crops increased with increasing N rate, but the fat content declined.

In case of the treatment D, the grain quality traits varied by growing season and year. In 2019, no difference in the starch content was observed in both main and ratoon crops. The protein content increased in the main crop with increasing D treatment but decreased in the ratoon crop. The fat content decreased in the main crop with increasing D treatment but increased in the ratoon crop. The tannin content increased in the main crop with increasing D treatment, but there was no difference in the ratoon crop. In 2020, the highest starch content was observed under D1 treatment in the main crop but under D2 treatment in the ratoon crop. The protein content of both main and ratoon crops showed the highest value under D2 treatment. In the main crop, the fat content decreased with increasing D treatment, but the tannin content increased. There were no differences in both fat and tannin contents in the ratoon crop.

## Discussion

### The Yield of Main and Ratoon Crops

It is generally believed that the agronomic and reproductive characters of ratoon sorghum reduced compared to the main crop (Mourtzinis et al., 2016; Ardiyanti et al., 2019). In our study, the yield of the ratoon crop was significantly lower than that of the main crop, which might be due to smaller SPAD, LAI, TDW, and greater reductions of SPAD and LAI from flowering to mature stage in the main season. Similar results were reported in previous studies on forage sorghum, sweet sorghum (Rao et al., 2011; Vinutha et al., 2017). There are many factors leading to this difference, including greater insect, disease, and weed damages on the ratoon crop than on the main crop (Duncan and Gardner, 1984; Gerik et al., 1988). Under suitable environmental conditions and appropriate cultivations, the yield potential of ratoon sorghum could be fully exploited (Al-Taweel et al., 2020). Afzal et al. (2012) suggested that a higher level of N application was needed to prevent production differences between main and ratoon crops of sorghum. In this study, with increasing N, the yield gap between main and ratoon crops narrowed.

The N application could enhance biomass and grain yield of sorghum significantly (Wortmann et al., 2007; Kaizzi et al., 2012). Our study indicated that a high N rate increased the grain yield of both main and ratoon crops, and the effects of N on yield and yield attributes of ratoon crops were greater than that of the main crop. Previous experiments on single-crop sorghum revealed that 150–225 kg N ha^–1^ was recommended for a high yield of waxy sorghum variety (Liang et al., 2017; Wang J. S. et al., 2017). Nevertheless, in our previous study (Zhou et al., 2021), 120 kg N ha^–1^ application only in the main season, same as N1 in this study, was enough to promote buds in stubble emerging and ratoon sorghum yield formation. Therefore, we considered that the recommended N rate exceeded the need for single-crop sorghum, and the N surplus could promote the growth of ratoon sorghum. Also, in view of the fact that N fertilizer had a larger effect on the grain yield of the ratoon crop than that of the main crop, we suggest a postponed and reduced N application in the main season. The N rate of 120 kg ha^–1^ is enough for the main crop, and to improve ratoon sorghum yield, a small amount of N is recommended top-dressed 2 weeks after the harvesting of the main crop.

With the increment of D, the SPAD of both main and ratoon crops declined, while LAI increased. The higher D caused more leaf cover and an increase in LAI (Mahmood et al., 2013), leading to a higher light interception but less chlorophyll content (Xiao et al., 2018). The responses of yield, TGW, and GWPE to D were the same between the main and ratoon crops, in line with previous research on single-crop sorghum (Alderfasi et al., 2016; Sahu et al., 2018), whose results showed that increasing planting densities led to raising biomass production and seed yield per unit area but low number and grain weight per panicle. An increase in the spikes per unit area resulting from a high number of plants per unit area compensated for the reduction in TGW and GWPE and produced a higher yield in higher density. However, beyond a certain density range, the grain yield of sorghum would decline (Yang et al., 2013; Zhou et al., 2016). In this study, the yield increased continuously with increasing D, indicating a higher density should be adopted for a higher yield, especially for ratoon crops with less dry biomass (Vinutha et al., 2017), LAI, and PH (Wang Y. C. et al., 2019). The density of over 127,500 plant ha^–1^ was recommended for the main crop, and a higher density for the ratoon crop could be reached by leaving three new buds on stubble in each hill.

### Grain Quality of Main and Ratoon Crops

Waxy sorghum is the best raw material for brewing liquor in China, especially famous liquor including Maotai, Wuliangye, Fenjiu, and so on (Liu et al., 2012). Starch is the main material to produce alcohol, and theoretically, the higher the starch content, the higher the liquor yield is. The protein hydrolyzes into amino acids, which can promote the growth of fermentation microorganisms and generate flavor substances to enhance the liquor taste (Yan et al., 2017). However, overmuch amino acids are harmful to liquor quality due to excess fusel oil; hence, an 8–10% protein content of sorghum grain was recommended for brewing (Tang, 2000). Fat is unfavorable to liquor-making, causing a fast and abundant acid generation, low liquor yield, and poor taste (Li, 1990). Tannin, suitable with a range of 0.5–2.0%, can inhibit harmful microorganisms and produce phenols in the fermentation process (Chen et al., 2012).

In this study, the ratoon crop recorded higher starch and tannin content but lower fat content compared with the main crop, indicating a better quality of ratoon crop. The quality of forage and sweet sorghum also showed differences between the main and ratoon crops (Rao et al., 2011; Vinutha et al., 2017). In terms of rice ratooning, extensive research has confirmed a higher rice quality of ratoon crop (Liu et al., 2002; Wu et al., 2019), due to a lower chalkiness and white vitreous and a higher milled rice recovery than that of the main crop (Cai et al., 2019). Duan et al. (2018) considered that the lower temperature and larger diurnal temperature range during the filling stage might be the reasons for the better quality of grain in the ratoon crop. Li et al. (2009) reported that 19.5–20.5°C was a suitable daily mean temperature for starch accumulation. The effect of environment on fat content was greater than those of genotype and genotype × environment (Zhang G. X. et al., 2010), and the fat content increased with the increase of daily temperature (Qu et al., 2019). Both high and low temperature stresses can promote the synthesis of plant tannin (Zhuang and Pan, 2008). The abovementioned research explained that the part of the reason for higher starch and tannin contents and lower fat content in ratoon sorghum may be lower daily temperature. As for the effects of cutting the main crop, depletion of nutrient levels in the soil and other meteorological factors for ratoon crop on quality differences in the main and ratoon crops, it is needed to conduct further experiments for confirmation.

The N fertilizer treatment is the key determinant among the three main fertilizers in effectively enhancing the quality of waxy sorghum (Wang C. et al., 2017). Appropriate N level can improve the capacity of carbon assimilation and N assimilation in the source organs, promote the translocation of assimilates from the vegetative organs to grains, and increase the activities of enzymes related to starch synthesis and N assimilation enzymes in grains, leading to the simultaneous increase of starch and protein content (Wang et al., 2003; Liu et al., 2017). In the process of grain filling, the synthesis pathways of starch and protein are synchronized, which are interdependent and competitive (Huppe and Turpin, 1994). It is generally believed that N fertilization increased grain protein content (Miao et al., 2006; Rashid et al., 2008), but high levels of N application might reduce starch content due to the reduced activities of enzymes involved in carbon assimilation and starch synthesis (Shen et al., 2006; Zhang X. L. et al., 2010). In this study, with increasing N rate, the starch content of the main crop decreased but that of the ratoon crop increased, while the protein content of both main and ratoon crops increased. The results indicated that after the consumption of N by the main crop, the residual N in the soil promoted the synthesis of starch and protein of the ratoon crop synchronously. Based on the abovementioned results, the yield improvement by N was mainly attributed to increased GWPE, and the increment of the ratoon crop was greater than that of the main crop. Starch is the main component of grain, whose content directly determines the grain weight. In the main crop, the starch content decreased with increasing N rate. It could be inferred that higher N input significantly enhanced spikelets per panicle, seed number per spike, and grain filling rate.

The D affected the photosynthetic rate and carbon assimilation ability of leaves by influencing the plant nutritional status and light distribution in the population (Sun et al., 2015). Most efforts to elucidate the effect of D on quality were focused on forage and sweet sorghum (Cavalaris et al., 2017; Sher et al., 2017), with less attention paid to grain sorghum. Available research results about the effect of D on grain quality of single-crop sorghum have not been consistent. Wang H. X. et al. (2016) reported that the starch content of sorghum grain decreased with increasing D, similarly with the result of our study on the main crop in 2020, while Wang C. et al. (2019) reported that the starch content first increased and then decreased. The protein content was not significantly influenced by D (Soleymani et al., 2011) or first increased and then decreased with the increase of density (Wang C. et al., 2019), which was consistent with our results on both main and ratoon crops. Although the protein content varied with N and D variation, it all met the demand of liquor production, so more attention should be paid to the effect of treatment on starch content and yield.

Previous studies showed that the effect of N application on fat content varies among the varieties (Yang et al., 2015), but the effect of D on fat content has not been reported. In our study, the variations of fat content according to N and D treatment were remarkably less and all lower 4%, meeting the criteria of raw materials for liquor production. Some reports showed that the effect of N on tannin content was not significant (Yin et al., 1990; Liu et al., 2017). Wang H. X. et al. (2016) reported that tannin content increased with increasing D, while Wang C. et al. (2019) demonstrated that tannin content first decreased and then increased. In this study, the tannin content increased with increasing N in both main and ratoon crops but increased with increasing D only in the main crop.

Compared with N and D treatments, growing season had a larger effect on sorghum grain quality. Therefore, it must be of great significance to further study the influence of growing season on quality parameters and why the influence occurred.

## Conclusion

The yield of the main crop was significantly higher than that of the ratoon crop. Increasing N fertilizer could increase the TGW, GWPE, and yield of both main and ratoon sorghum and could narrow the yield gap between the main and ratoon crop. N fertilizer had a larger effect on the grain yield of the ratoon crop than that of the main crop. Increasing D led to lower TGW and GWPE but higher grain yield. The ratoon crop recorded higher starch and tannin content but lower fat content compared with the main crop, indicating a better quality of ratoon crop. Compared with the growing season, N and D treatments had a smaller effect on sorghum grain quality. The simultaneous improvement of yield and quality can be obtained by increasing the yield of ratoon crops. Our results suggest that 120 kg N ha^–1^ in the main crop and a small amount of N top-dressed 2 weeks after main crop harvesting could obtain a high ratoon yield. A density of over 127,500 plant ha^–1^ is recommended for the main crop, and a higher density is suitable for the ratoon crop.

## Data Availability Statement

The original contributions presented in the study are included in the article/supplementary material, further inquiries can be directed to the corresponding author/s.

## Author Contributions

YZho and JH performed the experiments, analyzed data, and wrote the manuscript. ZL and YW carried out the experiment. JZ and YZha designed the experiments and revised the manuscript. All authors contributed to the article and approved the submitted version.

## Conflict of Interest

The authors declare that the research was conducted in the absence of any commercial or financial relationships that could be construed as a potential conflict of interest.

## Publisher’s Note

All claims expressed in this article are solely those of the authors and do not necessarily represent those of their affiliated organizations, or those of the publisher, the editors and the reviewers. Any product that may be evaluated in this article, or claim that may be made by its manufacturer, is not guaranteed or endorsed by the publisher.

## Footnotes

1
http://www.fao.faostat/en/#data/QC
